# Thiolation and characterization of regenerated *Bombyx mori* silk fibroin films with reduced glutathione

**DOI:** 10.1186/s13065-019-0583-x

**Published:** 2019-05-10

**Authors:** Xiaoning Zhang, Hong Bao, Carrie Donley, Jianwei Liang, Sha Yang, Shui Xu

**Affiliations:** 1 State Key Laboratory of Silkworm Genome Biology, College of Biotechnology, Southwest University, Chongqing, 400715 China; 2 Chapel Hill Analytical and Nanofabrication Laboratory, Department of Applied Physical Sciences, University of North Carolina at Chapel Hill, Chapel Hill, North Carolina 27599-3216 USA

**Keywords:** *Bombyxmori* silk, Silk fibroin film, Reduced glutathione, Surface modification, Cytotoxicity

## Abstract

*Bombyx* *mori* silk fibroin-based materials have good biocompatibility and biodegradability. In order to maximize their utility while maintain appropriate features, silk fibroin (SF) films were modified with reduced glutathione (GSH) (NH_2_)–ECG–(COOH), using the carbodiimide chemistry method, for the introduction of thiol groups onto surfaces. The effects of this modification on SF films’ chemical and physical properties, and cytotoxicity were assessed. The chemical and elemental composition analysis results suggested that reduced glutathione (GSH) was covalently coupled onto the surface of silk fibroin films. Atomic force microscopy (AFM) results indicated the surface roughness of silk fibroin film was increased after the modification by GSH. The GSH-modified silk fibroin films also showed the smaller contact angle due to the hydrophilic peptides coupled on the film surface. Through MTT assay, it was shown that the chemically modified SF film was not cytotoxic to HEK293 cells, and it had no adverse influence on the growth of HEK293 cells. Our approach provides a new option to engineer SF-based material surface with thiol groups in order to allow for secondary reactions and holds great promise for applications of SF-based materials in the biomedical field.

**Electronic supplementary material:**

The online version of this article (10.1186/s13065-019-0583-x) contains supplementary material, which is available to authorized users.

## Introduction

Natural biopolymers could be used in various biomedical applications [1, 2]. As one of natural biopolymers, silk secreted by silkworm *Bombyx* *mori* consists of silk fibroin (SF) and sericin. The two paralleled silk fibroin fibers are held together with a layer of sericin on their surface. Among SF and sericin [3], SF have good biocompatibility, and have shown its potential to be used as substitute materials for bone, cartilage and ligaments [4–6]. In addition, SF materials have good biodegradability. The degradation products of SF are not only non-toxic, but are also helpful for nutrition and repair of tissues such as skin and periodontal tissues [7]. Other advantages of SF materials include the ability to withstand sterilization conditions without losing their integrity, easy to prepare, and stable at room temperature [8]. Therefore, SF materials have been widely used in tissue engineering [9, 10].

Chemical modification plays an important role in the application of SF materials as it expands the utility of this protein family. Within 5000 amino acids of silk fibroin, some of them, such as serine, threonine, aspartic acid, glutamic acid, and tyrosine, can be modified with known chemistries [11]. A variety of silk fibroin modification chemistries have been reported. Those approaches are applicable to aqueous forms of SF (in the pre-formulation stage) or solid form of SF (surface modification only and leaving the material bulk intact) [12]. Among various strategies, carbodiimide chemistry has been widely used as it is applicable to both solid and aqueous forms of SF.

Thiolation is an effective way to allow formation of functionalities of materials, as thiol groups can serve as crosslinkers to further tune the surface chemistry of materials [13–15]. Glutathione (GSH) is formed by peptide bond condensation of glutamic acid, cysteine and glycine. This tripeptide is the principal intracellular non-protein thiol in eukaryotes and some prokaryotes [16]. Here, we demonstrate a chemical modification strategy to tailor the surface property of SF film by introducing functional handles of thiol groups from GSH, in order to allow for secondary reactions. In our work, silver modified SF film was prepared through the formation of covalent bonds between thiol groups and silver. The antimicrobial activity of SF/silver composite film was excellent. Therefore, the SF film functionalized with GSH has proven to held great promise for applications in the biomedical field, such as wound dressing. The obtained composite films were morphologically and structurally characterized. In addition, initial studies into the biocompatibility of these modified SF films were assessed in the present work.

## Materials and methods

### Reagents and chemicals

Fresh cocoons of *Bombyx* *mori* silkworm were obtained from College of Biotechnology, Southwest University. Both sodium carbonate anhydrous (Na_2_CO_3_) and calcium chloride anhydrous (CaCl_2_) were purchased from Kelong chemical reagent factory (Chengdu, China) and at AR grade. Anhydrous ethanol (C_2_H_6_O, AR grade) was purchased from Chongqing Chuandong chemical group Co. (Chongqing, China). 1-ethyl-3-(dimethylaminopropyl) carbodiimide hydrochloride (EDC) and *N*-hydroxysuccinimide (NHS) were purchased from Solarbio (Beijing, China). DMEM (high glucose) and PBS (1×) were purchased from Hyclone (Utah, United States). Ultrapure water (resistance > 18 MΩ cm^−1^) was used in all experiments.

### Preparation of SF solution and SF films

**Fig. 1:**
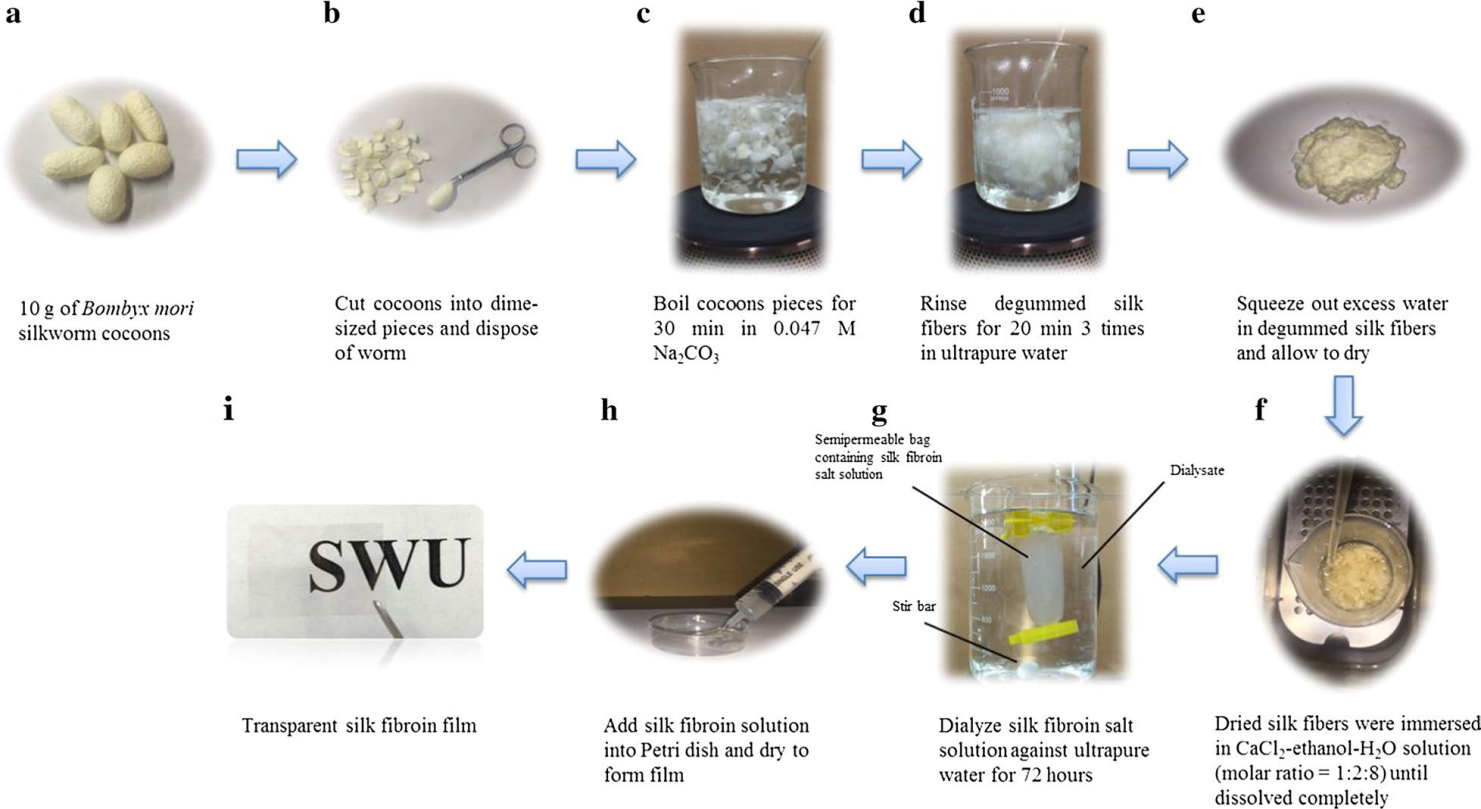
Schematic for making silk fibroin film

Silk fibroin solution was prepared (Fig. 1a–g), and its concentration was determined as previously described by Kaplan et al. [17]. The silk fibroin solution was then concentrated slowly at 60 °C to a final concentration of 6 wt%.

To prepare silk fibroin films, 2.5 mL of 6% aqueous silk fibroin solution was added into a 60 mm Petri dish and allow it to dry overnight without covering the Petri dish (Fig. 1h). Once dried, the films were water-annealed by placing samples within a water filled desiccator and with a 25 kPa vacuum for 12 h. This water-annealing technique produces water-insoluble films [18]. Each SF film sample was then removed from the Petri dish. The films produced by this method have excellent transparency (Fig. 1i).

### Covalent coupling of GSH to SF films

**Scheme 1:**
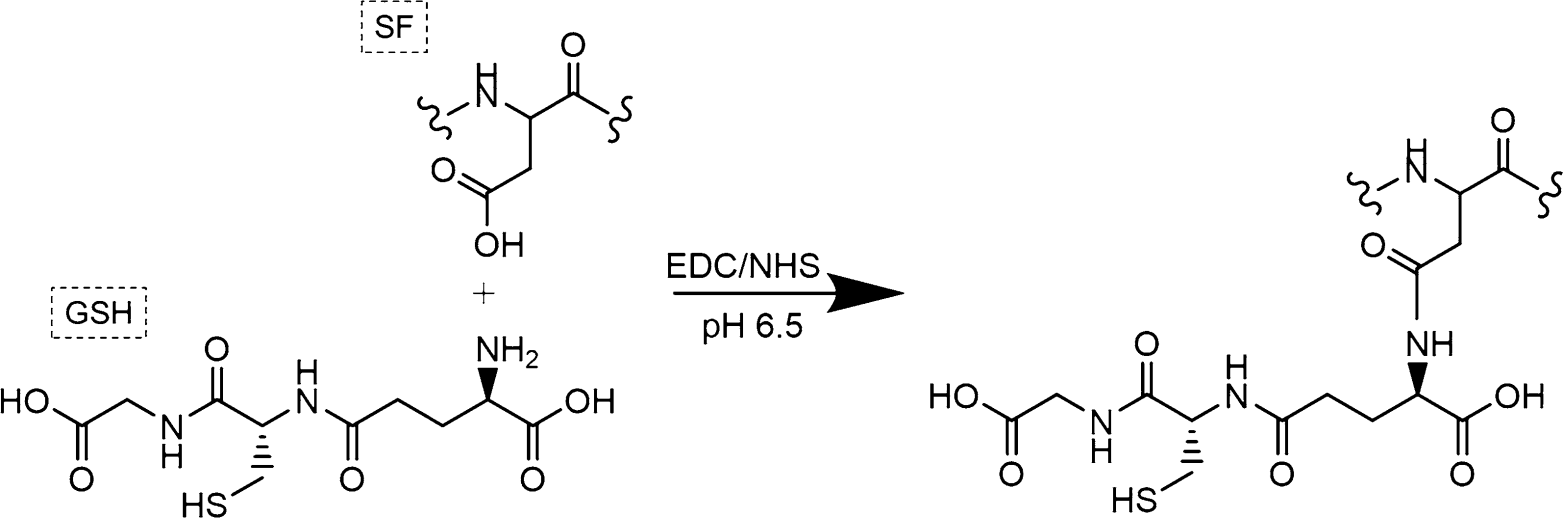
Carbodiimide coupling between SF and GSH

The prepared silk fibroin films were soaked in PBS buffer (pH 6.5) for 30 min, in order to hydrate the films and induce any surface rearrangement to bring forth hydrophilic functional groups. The PBS buffer solution was then replaced by 1-ethyl-3-(dimethylaminopropyl) carbodiimide hydrochloride (EDC)/*N*-hydroxysuccinimide (NHS) solution (0.5 mg/mL of EDC with 0.7 mg/mL NHS in PBS buffer) to activate the –COOH groups from aspartic and glutamic acids (15 min at room temperature). After activation with EDC/NHS, the SF films were rinsed with PBS buffer (pH 6.5) for 5 min, and then contacted with 0.1 mg/mL GSH in PBS buffer (pH 6.5) for 2 h at room temperature. The activated carboxyl group is very reactive towards the primary amines on the GSH, therefore linking the carboxyl groups on the silk fibroin film surface and the amine groups of GSH molecules, or vice versa, together, and forming stable amide bonds between GSH and the silk fibroin molecules (Scheme 1) [19]. At the completion of peptide coupling, the films were rinsed for 5 min in fresh PBS buffer (pH 6.5) solution three times, and then rinsed twice with ultrapure water to remove unbound peptide and buffer salts remains prior to use for next step.

### Surface characterization of SF films

X-ray photoelectron spectroscopies (XPS) were recorded on a Kratos Axis Ultra DLD X-ray Photoelectron Spectrometer (Kratos Analytical Ltd., United Kingdom) in an ultrahigh-vacuum system with a base pressure of 5 × 10^−9^ Torr, a monochromatic Al K*α* source, and a hemispherical analyzer. Survey (1 eV resolution) and high-resolution (0.1 eV resolution) spectra were collected at a 0° take off angle from surface normal. Each high-resolution spectrum was referenced with respect to the 284.6 eV C 1s level observed to eliminate charge effects and fit with Voigt functions (70% Gaussian, 30% Lorentzian) after a Shirley background correction.

The FT-IR spectra of silk fibroin films were recorded on a Nicolet IS5 attenuated total reflection Fourier transform infrared (ATR-FTIR) instrument (Thermo Fisher Scientific, USA) in the range of 800–4000 cm^−1^ with a resolution of 4 cm^−1^. Each spectrum represents 32 co-added ratioed against a reference spectrum obtained by recording 32 co-added scans of an empty ATR cell.

Static water contact angles of the silk fibroin films were measured by dropping 1 μL of deionized water onto the silk fibroin film surfaces using a KRUSS Contact Angle Analyzer (Kruss GmbH, Germany) at 25 °C. The measurement of each contact angle was made within 10 s after each drop. Three films of each type were analyzed, and each film was measured in at least three areas. Smaller contact angles correspond to increased wettability.

The surface morphology of SF films was characterized on an Asylum Research MFP3D AFM in air at ambient conditions. Tapping mode measurements were acquired with an aluminum reflex-coated silicon cantilever (force constant of 40 N/m) at a resonant frequency of 300 kHz. Reported roughness values are the average and standard deviation of three separate 2 × 2 μm^2^ regions on the same surface. The software used for image processing was WSxM [20].

### X-ray diffraction

X-ray diffraction was performed by a Shimadzu XRD-6100 X-ray Diffractometer with Cu-Kα radiation (λ = 0.15406 nm). The voltage of the X-ray source was 30 kV at a current of 20 mA. The diffraction intensity curves were obtained at a scanning rate of 4°/min and within the scanning region of 2*θ* = 5−50°.

### Cytotoxicity assay

After sterilization by UV light for 30 min, 0.2 g of each sample was cut into small pieces in a laminar air flow cabinet (Suzhou Antai Airtech Co., Ltd, China), and were immersed in 10 mL sterilized Dulbecco’s modified Eagles medium (DMEM) respectively. HEK293 cells, a kind of epithelial cell, were used in cytotoxicity studies. Cells were plated at a density of 5 × 10^3^ cells per well in 200 μL DMEM with 10% (v/v) fetal bovine serum (FBS). Cells were maintained at 37 °C in a 95% air, 5% CO_2_ atmosphere. The cells were first incubated for 24 h, and then 20 μL of leaching liquor with different concentration was added into each well. 24 h, 48 h, and 72 h later, 20 μL of 3-(4,5-dimethyldiazol-2-yl)-2,5-diphenyltetrazolium bromide (MTT) solution (5 mg/mL in PBS) was added into each well for a 4-h incubation. After the removal of culture medium, cells in each well were then lysed in 150 μL of dimethyl sulfoxide (DMSO) for 10 min. Optical density (OD) was measured on a microplate reader (Synergy H1 Hybrid Multi-Mode Reader, Gene Company Limited) at the wavelength of 490 nm.

The cells cultured without leaching liquor treatment were used as control. The average value of three parallel experiments was collected and the cell viability was calculated via the following equation [21]:

$${\text{Cell viability }} ( \% ) = {{\text{ OD}}_{490}}\, \left( {{\text{sample}}} \right)/{{\text{OD}}_{490}} \,\left( {{\text{control}}} \right) \times 100\%$$

### SF film cell culture system setup

SF films were dipped into 24-well plate prefilled with 1 mL of 70% ethanol solution for 10 min to ensure sterility. Then, 70% ethanol solution was removed, and each sample was washed three times with 1 mL of PBS solution (pH 6.5, let each wash sit for 5 min to allow for complete diffusion). After removal of PBS solution, 500 μL of HEK293 cells suspension (5 × 10^3^ cells) was sampled in each well. The cultures were placed within incubator (SANYO Electric Biomedical Co., Ltd., Japan) at 37 °C and 5% CO_2_ for 3 days, and the cell growth morphology was observed under a XDS-1B inverted microscope (Chongqing, China).

## Results and discussion

### ATR-FTIR analysis of SF films

**Fig. 2:**
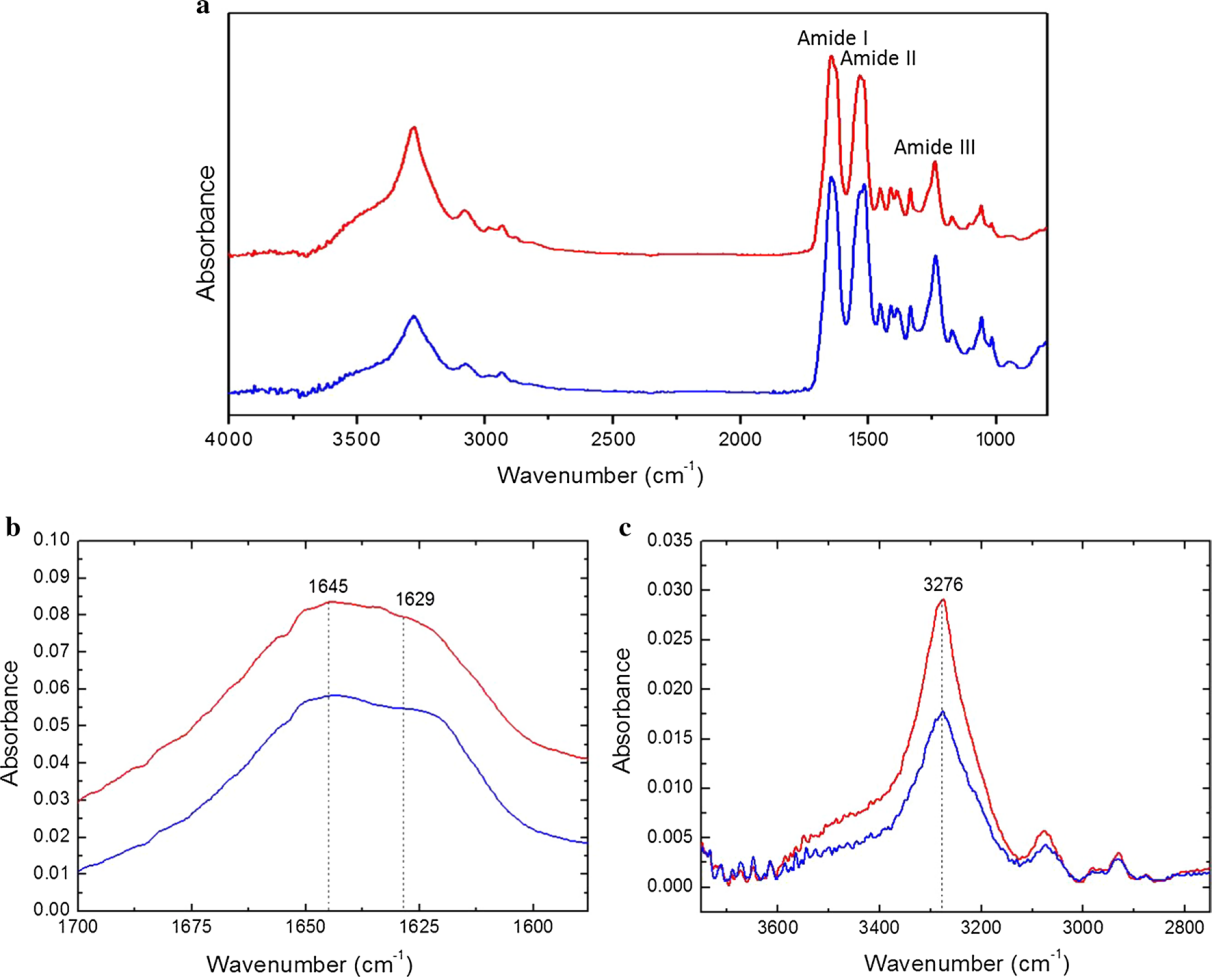
**a** FTIR spectra of the silk fibroin film before (blue line) and after (red line) the carbodiimide coupling reaction; **b** zoom-in of (**a**) for the region from 1588 cm^−1^ to 1700 cm^−1^; **c** zoom-in of (**a**) for the region from 2750 cm^−1^ to 3750 cm^−1^

Infrared absorption spectra of water annealed and GSH modified SF film show characteristic absorption bands assigned to the peptide bonds (–CONH–) that originate bands known as amide I, amide II, and amide III (Fig. 2a). It can be observed from FTIR spectra that amide I, amide II, and amide III of both water annealed and GSH grafted SF film were at 1645 cm^−1^, 1522 cm^−1^, and 1235 cm^−1^, respectively. Therefore, it can be concluded that GSH modification had no significant effect on the secondary structure of the silk fibroin film.

In addition, amide I is useful for the analysis of the secondary structure of the proteins and is mainly related with the C=O stretching, and it occurs in the range of 1700–1600 cm^−1^. The water annealed SF film spectra shows a peak at 1645 cm^−1^, suggesting the presence of the helical conformation [22], with a shoulder at about 1629 cm^−1^, indicating a level of *β*-sheet conformation [23], for amide I. FTIR spectra of GSH-modified SF film showed a peak at 1645 cm^−1^ with a strong shoulder at about 1629 cm^−1^ for amide I as well, which reflects a similar molecular conformation as that of water annealed SF film (Fig. 2b).

FTIR spectroscopy also provides evidence that GSH was grafted onto SF film. The absorption band at 3276 cm^−1^ represents for N–H stretching vibration of amide [24], and the intensity of this band after carbodiimide coupling is apparently increased indicating the increased density of the amide bond (Fig. 2c), which is from the reaction between primary amine of reduced glutathione (GSH) and activated carboxylic groups in silk fibroin, or vice versa.

### XPS analysis of SF films

**Fig. 3:**
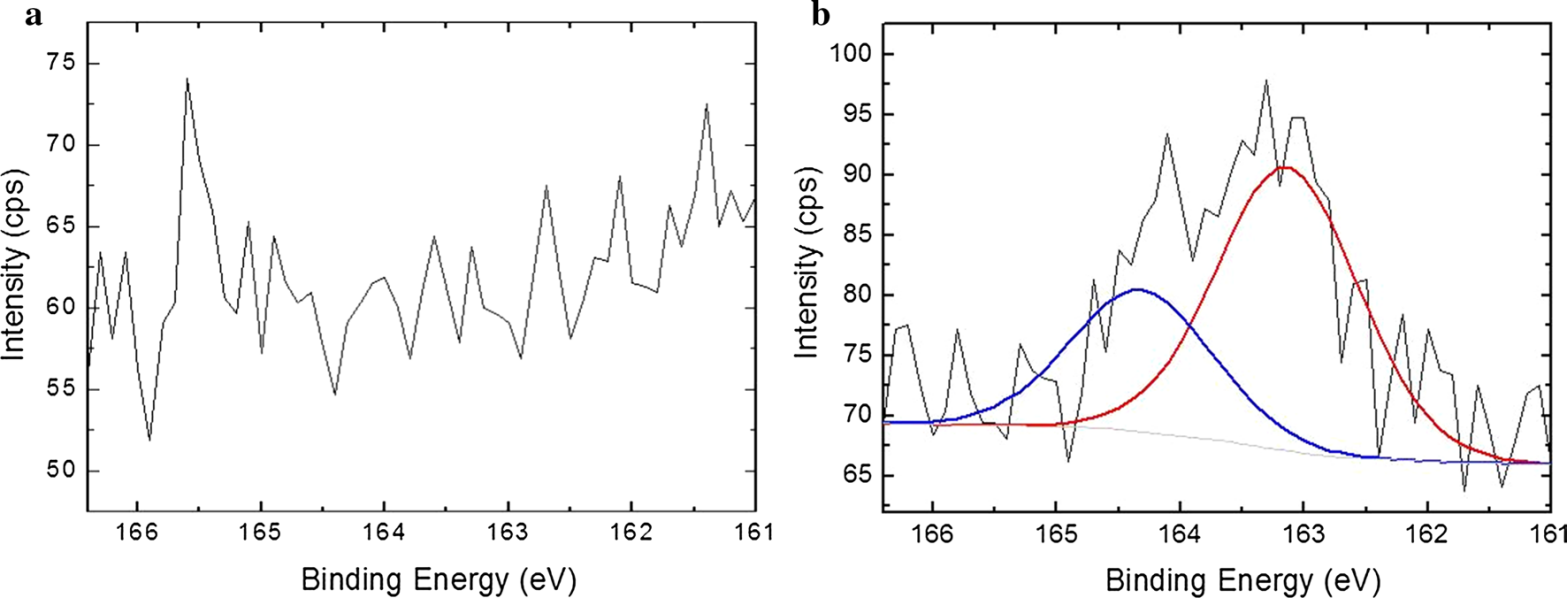
High-resolution XPS spectra of the S 2p region measured from a silk fibroin film surface before (**a**) and after (**b**) the carbodiimide coupling reaction with GSH. Raw data are represented with a solid black line, and individual Voigt-fit components are represented with solid colored lines

Figure 3 showed XPS analysis results of the surface S 2p high resolution spectra of a water annealed (Fig. 3a) and a GSH-modified (Fig. 3b) SF films. Compared with water annealed sample, a new peak for the elemental 2p sulfur was detected on the surface of GSH-modified one. It can thus be concluded that sulfur component is present on the surface of SF films, therefore confirmed the successful installation of GSH on the surface of SF films. Deconvolution of XPS S 2p spectra was performed to estimate chemical state of thiol groups. A typical curve fitting of the S 2p peak region is shown in Fig. 3b. The doublet at 163.1 eV is consistent with the formation of disulfide bonds [25]. Therefore, a disulfide bond reducing agent is required in order to access the free thiol groups on SF film.

**Fig. 4:**
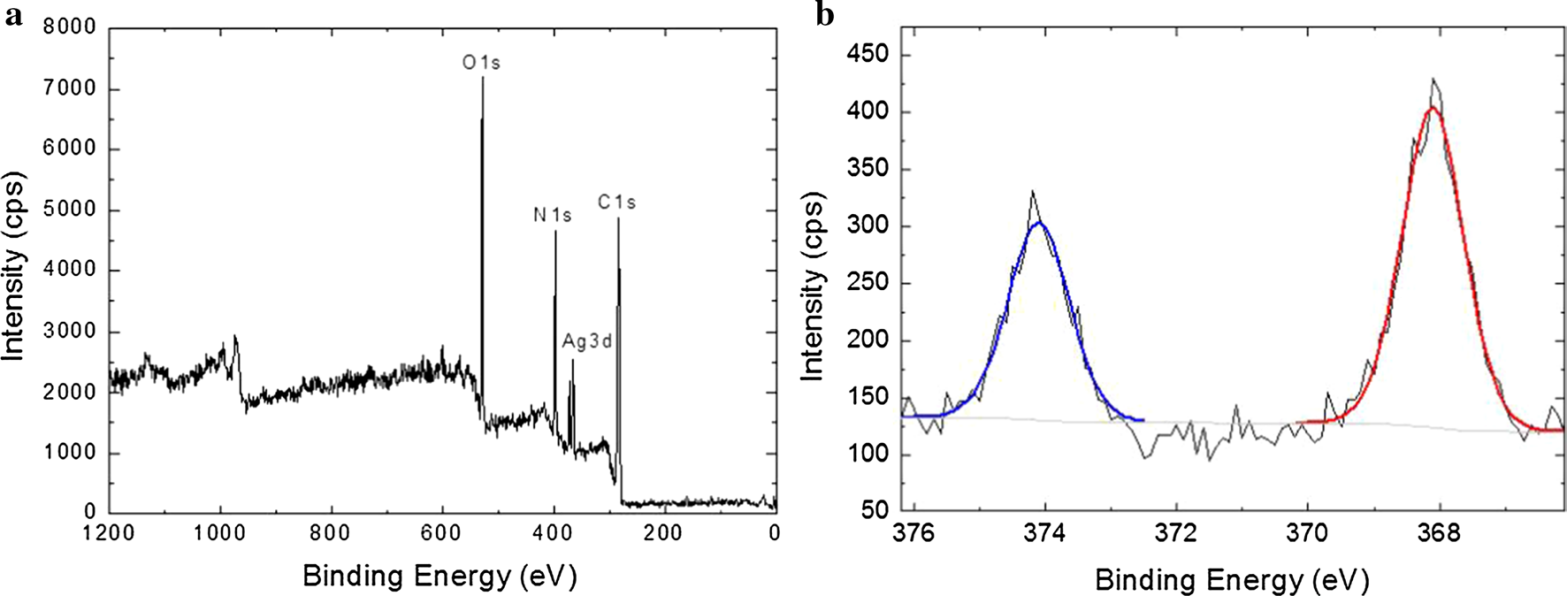
XPS survey spectrum of Ag-loaded silk fibroin film (**a**). High-resolution XPS spectrum of the Ag 3d region measured from an Ag-loaded silk fibroin film (**b**). Raw data are represented with a solid black line, and individual Voigt-fit components are represented with solid colored lines

In order to confirm the reactivity of thiol groups on GSH-modified silk fibroin film, the sample was kept in 10 mM TCEP·HCl solution for 20 min at room temperature followed by rinsing with ultrapure water thoroughly. The sample was then incubated in 10 mM AgNO_3_ solution for 10 min, followed by sonicating in ultrapure water for 5 min to remove bound AgNO_3_ and rinsed thoroughly with ultrapure water. Figure 4a shows the XPS survey spectra of GSH-modified SF film after soaking in AgNO_3_ solution, where the typical peaks assigned to C 1s, N 1s and O 1s can be observed at 285 eV, 400 eV and 532 eV clearly [26]. The results of the XPS spectrum in Fig. 4a also confirmed the existence of Ag (368 eV) on the sample surface, suggesting the existence of interactions between SF and Ag. Then, a high-resolution XPS spectrum of Ag 3d was recorded. The core level binding energy of Ag 3d was observed as 374.2 and 368.2 eV which is attributed to the Cys-capped Ag [27]. This means that Ag was linked to the Cys of GSH grafted onto the silk fibroin film surface, indicating the presence of metal–thiolate bonding and illustrating the reactivity of the HS-SF film. In addition, the potential use of Ag/SF film composite as antibacterial material was assessed by observing their antibacterial activity against *E. coli*. The antibacterial properties of silver-loaded SF films were evaluated by disc diffusion assay. It was found that silver-loaded SF films can effectively inhibit the growth of *E. coli* (Additional file 1: Figure S1).

### Hydrophobicity

**Fig. 5:**
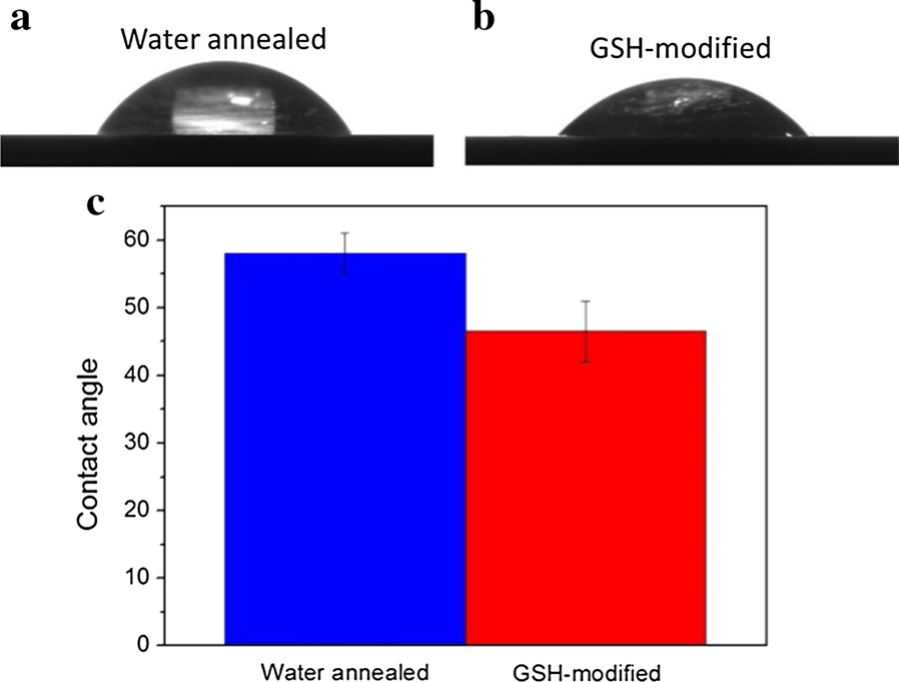
Representative static contact angles of 1 µL drops of ultrapure water resting on SF films before (**a**) and after GSH modification (**b**)

The contact angles measured on the water annealed silk fibroin films and the GSH-modified silk fibroin films were presented in Fig. 5. Smaller contact angle usually indicates that the material surface is more hydrophilic, enhancing the cell adhesion and proliferation. The contact angle for the water annealed silk fibroin film was 57.9 ± 3.0°. A significant difference (*p* < 0.05) was detected after the water annealed silk fibroin film was treated with carbodiimide coupling reaction, which had the contact angle of 46.5 ± 4.4°. Compared to the water-annealed silk fibroin films, the decrease of contact angle may be attributed to the covalent coupling of hydrophilic peptides.

### Morphology

**Fig. 6:**
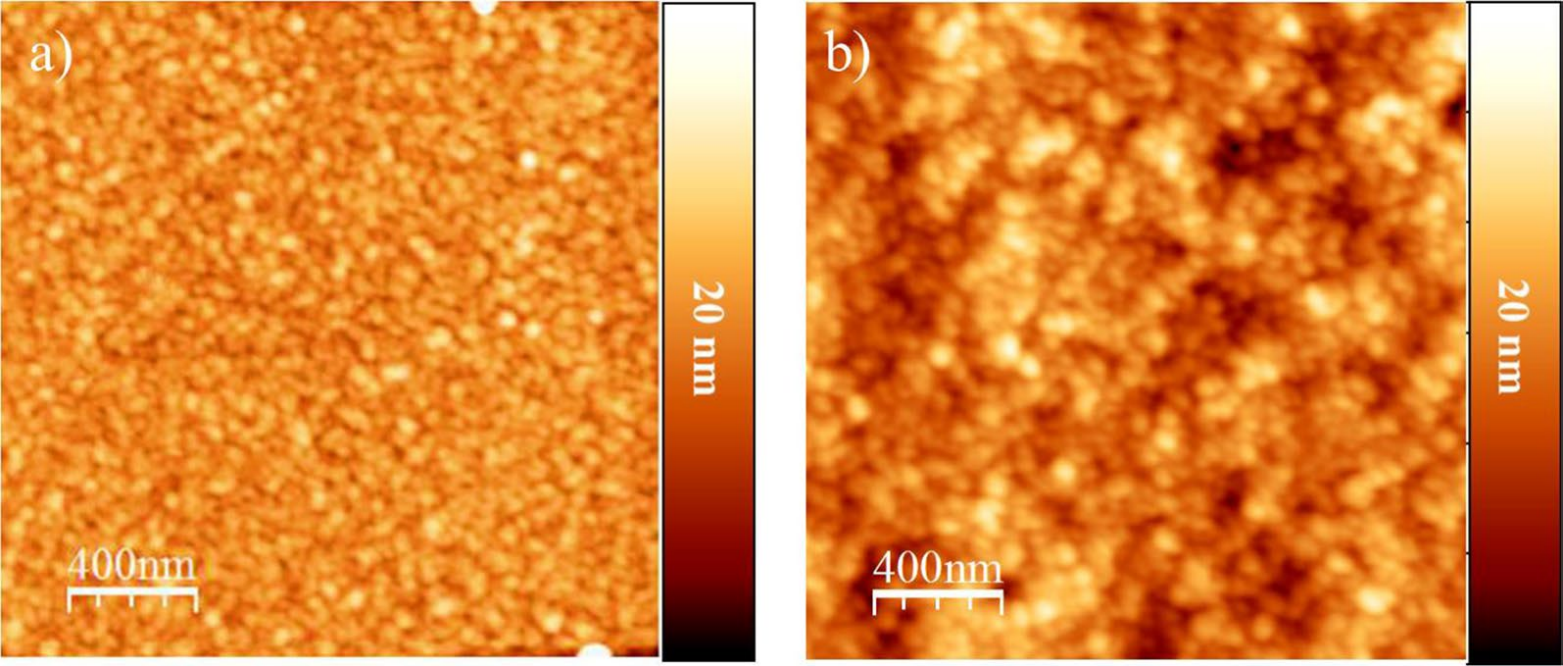
Topographies of AFM images (tapping mode) for SF films before (**a**) and after GSH modification (**b**)

AFM was used to exam surface morphologies of samples, and root-mean-square (RMS) values were calculated to determine surface roughness of samples. For water annealed SF film, granular features with a lateral dimension of 49.2 ± 3.6 nm totally covered its surface (Fig. 6a). Those aggregated granules were densely grouped together. GSH-modified SF film was very similar to water annealed SF film but the granules were much larger than those formed on the surface of water annealed SF film with a lateral dimension of 59.2 ± 4.7 nm (Fig. 6b). The surface roughness was increased from 1.6 ± 0.1 to 2.4 ± 0.3 nm (n = 3). This result suggested that the peptide covalent coupling to the surface of SF films increases their surface roughness.

### Structure analysis

**Fig. 7:**
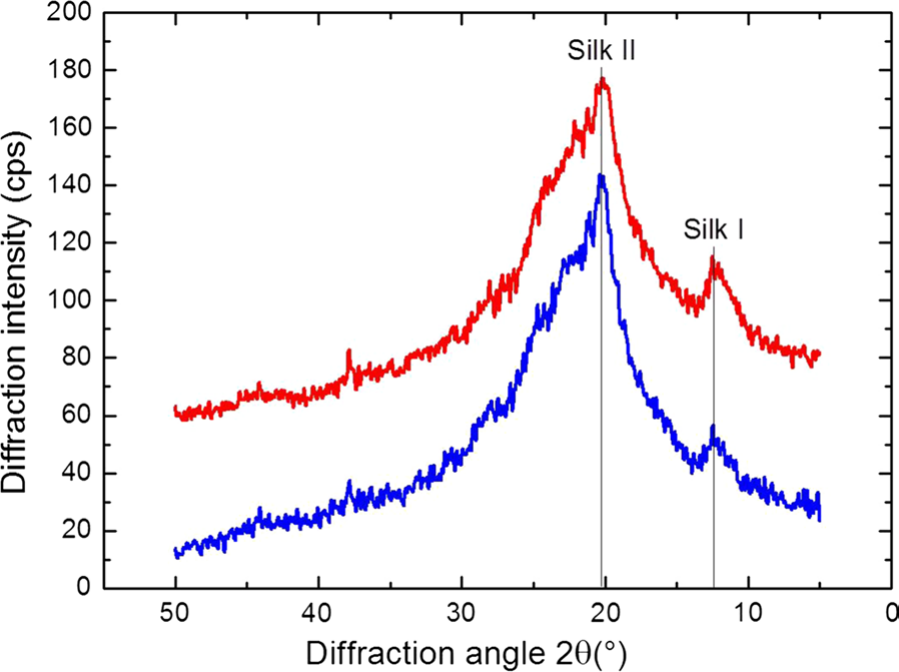
XRD profiles of the silk fibroin films before (blue line) and after (red line) the carbodiimide coupling reaction with GSH

To detect if changes in the crystalline structure were induced by carbodiimide coupling, X-ray diffractometer (XRD) profiles of the silk fibroin film before and after the carbodiimide coupling reaction were examined (Fig. 7). The principal diffraction peaks of the Silk I crystal structure (random coil content) are 12.2°, 19.7°, 24.7° and 28.2°; the diffraction peaks of Silk II crystal structure (*β*-sheet content) are 9.1°, 18.9° and 20.7° [28–30].

Both X-ray diffraction curves of water annealed and GSH-modified SF films show diffraction peaks at 2*θ* = 12.2° and 20.7°. Above data indicate some silk I structure and silk II structure are in these films. Silk I conformation can be explained by slow crystallization for silk fibroin from liquid during film casting at ambient conditions [31]. While water annealing process can gradually change SF film’s crystal structure from silk I to silk II [30]. No obvious change in the crystalline structure was observed from the X-ray diffraction of silk fibroin film after carbodiimide coupling reaction.

### Biocompatibility

The MTT assay is a common method for evaluating biomaterial toxicity based on the mitochondrial activity, which influences metabolic activity and cell viability. To determine the toxicity profile of the GSH-modified silk fibroin film, we conducted the standard MTT cytotoxicity assay with HEK293 cells [32, 33].

**Fig. 8:**
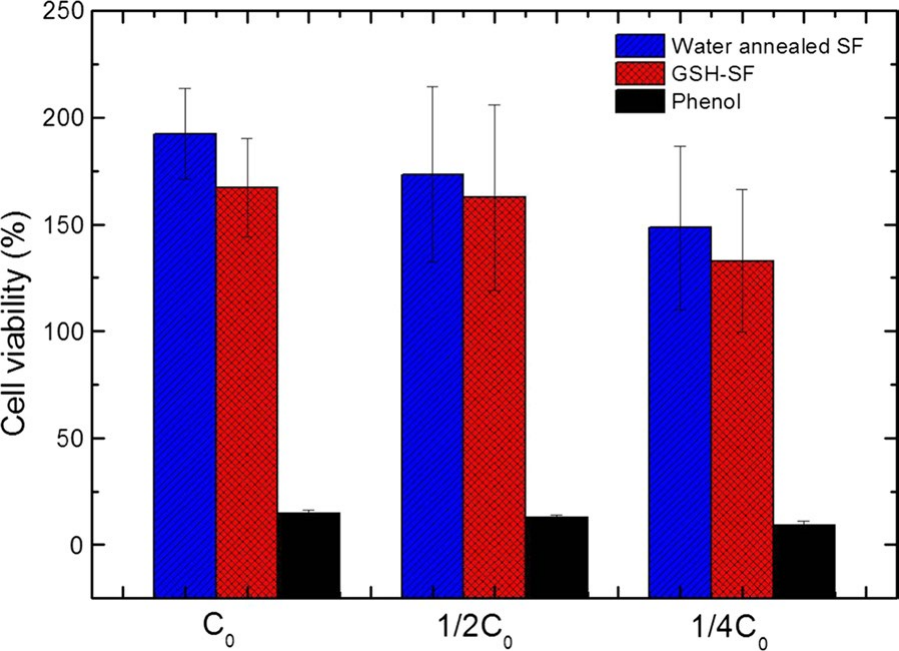
HEK293 cells viability in leaching liquor obtained from samples (water annealed and GSH-modified SF films) evaluated by MTT assay. Complete growth medium supplemented with 0.64% phenol served as a positive control (n = 3)

As Fig. 8 demonstrates, the cells were treated with leaching liquors from water annealed SF film and GSH-modified SF film, as well as phenol solution with various concentrations respectively, which were coded as C_0_, 1/2C_0_, and 1/4C_0_ (Fig. 8). Comparing the leaching liquor from water annealed SF film, the leaching liquor from GSH-modified SF film decreased the HEK293 cell viability slightly. This difference perhaps to be relative with the presence of EDC trace residue within the sample, and the previous study demonstrated that EDC had negative effects on the cell viability [34]. Apart from that, both water annealed and GSH-modified SF films exhibited high biocompatibility as their cell viabilities were higher than 100% relative to control. In addition, the HEK293 cell viability of both water annealed and GSH-modified SF films was slightly decreased after their leaching liquors were diluted. Because it is a dose dependent continuous decrease, we could conclude that silk fibroin based material can enhance cell proliferation.

**Fig. 9:**
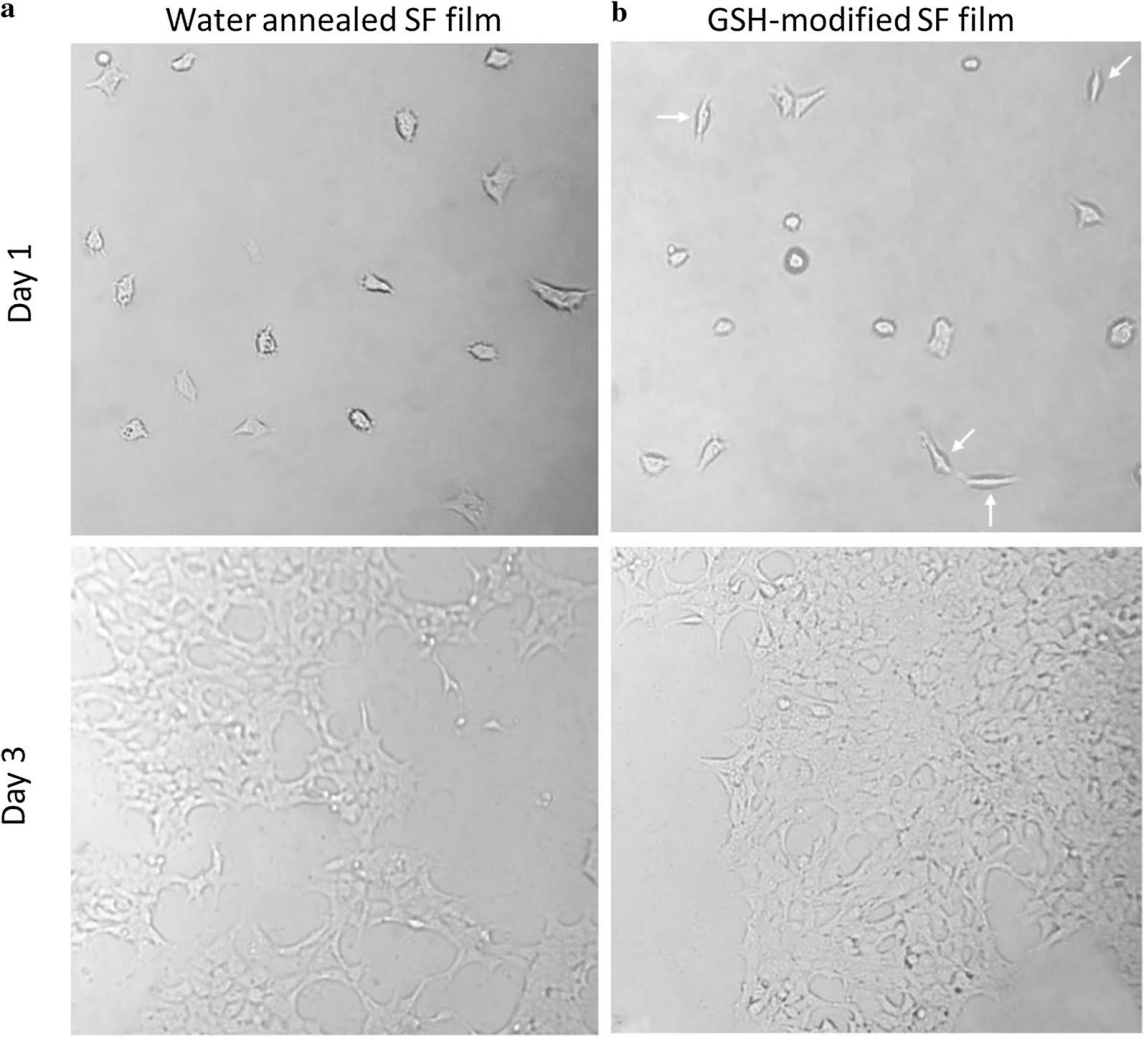
The morphologies (×200) of HEK293 cells on water annealed (**a**) and GSH-modified (**b**) SF films after seeding (1 and 3 days)

HEK293 cell adhesion on surfaces of water annealed and GSH modified SF films were observed to better understand how GSH modification can play a role in cell adhesion and spreading (Fig. 9).

At day 1, optical microscope image indicated that cell adhesion was randomly distributed on both water annealed and GSH modified SF films. In addition, certain number of cells appeared in an elongated shape to GSH modified SF film, but with a widening morphology, or very flat, on water annealed SF film. This might because a smoother surface (water annealed SF film) can help in the very first states of cell adhesion.

Three days after seeding, the cells that adhered to water annealed and GSH modified silk fibroin film were comparable both in terms of number and morphology. In addition, at this time, individual cells were in direct contact with other cells forming a monolayer. These data indicate that GSH-modified SF films are biocompatible and have no adverse influence on the growth of the HEK293 cells.

## Conclusions

In summary, we developed a simple method for grafting thiol moieties from the reduced glutathione onto the silk fibroin film through carbodiimide coupling reaction. The chemical and physical structures of GSH-modified SF film were characterized by FTIR, XPS, and XRD. The surface morphology of GSH-modified SF film was characterized by atomic force microscopy (AFM). The reactivity of the thiol groups was proved by conjugating to silver. In addition, the prepared GSH-modified silk fibroin film exhibited the excellent biocompatibility. As thiol groups can serve as crosslinkers for the covalent binding of functional molecules, in order to modify biomaterials, alone or through click reaction, we believed that the strategy used in this work to introduce thiol groups onto the surface of SF material has a great potential in expanding the applications of SF materials in drug carries, tissue scaffolds and implantable devices.

## Additional file

**Additional file 1: Figure S1.** Antibacterial test. Antibacterial property of water annealed, GSH-modified, and silver-loaded SF films against* E. coli*.

## Data Availability

The datasets used and analysed during the current study are available from the corresponding author on reasonable request.

## Abbreviations

GSH: glutathione
SF: silk fibroin
AFM: atomic force microscopy
EDC: 1-ethyl-3-(dimethylaminopropyl) carbodiimide hydrochloride
NHS: *N*-hydroxysuccinimide
XPS: X-ray photoelectron spectroscopies
ATR-FTIR: attenuated total reflection Fourier transform infrared
MTT: 3-(4,5-dimethyldiazol-2-yl)-2,5-diphenyltetrazolium bromide
DMEM: Dulbecco’s modified Eagles medium
PBS: phosphate-buffered saline
DMSO: dimethyl sulfoxide
FBS: fetal bovine serum
RMS: root-mean-square
XRD: X-ray diffractometer
