# Supplementary material for: Thiolation and characterization of regenerated Bombyx mori silk fibroin films with reduced glutathione
Source: BMC Chem. 2019 May 10;13(1):62. doi: 10.1186/s13065-019-0583-x (PMC6661838; doi:10.1186/s13065-019-0583-x)

**Additional File 1**

The antibacterial property of silver-loaded SF film was determined by disc diffusion assay, which is a conventional method to test the inhibitory effect of antibacterial agents [1-3]. In our study, silver-loaded SF films were cut into about 10-mm diameter discs under aseptic condition. The samples were rinsed with 70% ethanol and left to dry inside a laminar-flow hood. Each disc was then rehydrated with an 8 *μ*l drop of normal saline before being placed on LB agar plates inoculated 10^6^ colony forming unit (CFU)/ml of *E.coli*. After 18 h incubation at 37ºC, the inhibited areas of bacterial growth surrounding each sample was recorded by digital camera and measured (Fig. 1S). Silver-loaded SF film produced clear zones of inhibition on agar plates incubated with *E.coli* bacteria. While water annealed SF and GSH-modified SF did not produce any observable inhibition of *E.coli* growth. These data show that antibacterial properties can be conferred to GSH-modified SF films by loading them with Ag via Ag-S interaction.

**References**

1. Poggio C, Lombardini M, Colombo M, Dagna A, Saino E, Arciola CR, et al. Antibacterial effects of six endodontic sealers. International Journal of Artificial Organs. 2011;34:908-13.

2. Madsen J, Armes SP, Bertal K, Lomas H, MacNeil S, Lewis AL. Biocompatible wound dressings based on chemically degradable triblock copolymer hydrogels. Biomacromolecules. 2008;9:2265-75.

3. Percival SL, Slone W, Linton S, Okel T, Corum L, Thomas JG. The antimicrobial efficacy of a silver alginate dressing against a broad spectrum of clinically relevant wound isolates. International Wound Journal. 2011;8:237-43.

**Figure 1S** Antibacterial property of water annealed (a), GSH-modified (b), and silver-loaded (c) SF films against *E.coli*.

**Figure 1S.**


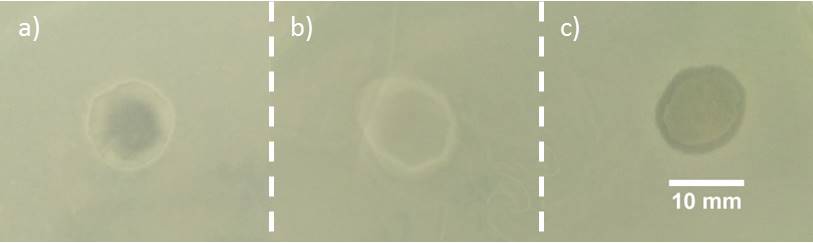

Supplement: Supplementary file 1 — Additional file 1: Figure S1. Antibacterial test. Antibacterial property of water annealed, GSH-modified, and silver-loaded SF films against E. coli. [file 13065_2019_583_MOESM1_ESM.docx]
